# Lipofundin MCT/LCT Inhibits Levcromakalim-Induced Vasodilation by Inhibiting Endothelial Nitric Oxide Release

**DOI:** 10.3390/ijms21051763

**Published:** 2020-03-04

**Authors:** Soo Hee Lee, Dawon Kang, Seong-Ho Ok, Ji-Yoon Kim, Sung Il Bae, Yeran Hwang, Kyeong-Eon Park, Jong Won Kim, Ju-Tae Sohn

**Affiliations:** 1 Department of Anesthesiology and Pain Medicine, Gyeongsang National University Hospital, 15 Jinju-daero 816 Beon-gil, Jinju-si 52727, Koreathesonkim@gmail.com (J.W.K.); 2 Department of Physiology, Gyeongsang National University School of Medicine, Jinju-si 52727, Korea; 3 Department of Anesthesiology and Pain Medicine, Gyeongsang National University Changwon Hospital, 11 Samjeongja-ro, Seongsan-gu, Changwon-si 51427, Korea; 4 Department of Anesthesiology and Pain Medicine, Gyeongsang National University College of Medicine, 15 Jinju-daero 816 Beon-gil, Jinju-si 52727, Korea; 5 Department of Anesthesiology and Pain Medicine, Gyeongsang National University College of Medicine, Gyeongsang National University Hospital, 15 Jinju-daero 816 Beon-gil, Jinju-si 52727, Korea; 6 Institute of Health Sciences, Gyeongsang National University, Jinju-si, 52727, Republic of Korea

**Keywords:** lipid emulsion, levcromakalim, nitric oxide, ATP-sensitive potassium channel

## Abstract

The goal of this study was to examine the effect of lipid emulsion on the vasodilation induced by ATP-sensitive potassium (K_ATP_) channels in isolated rat aortae and the underlying mechanism. The effects of Intralipid, containing 100% long-chain fatty acids, and Lipofundin MCT/LCT, containing 50% long-chain fatty acids plus 50% medium-chain fatty acids, on the vasodilation induced by levcromakalim in endothelium-intact aorta with or without N^W^-nitro-L-arginine methyl ester (L-NAME) and in endothelium-denuded aorta were examined. The effects of L-arginine, L-NAME, glibenclamide, and Lipofundin MCT/LCT, alone or combined, on the levcromakalim-induced vasodilation were examined. Lipofundin MCT/LCT inhibited the levcromakalim-induced vasodilation of isolated endothelium-intact aortae, whereas Intralipid did not. In addition, Lipofundin MCT/LCT had no effect on the levcromakalim-induced vasodilation of endothelium-denuded rat aortae and endothelium-intact aortae with L-NAME. L-arginine and Lipofundin MCT/LCT produced more levcromakalim-induced vasodilation than Lipofundin MCT/LCT alone. Glibenclamide inhibited levcromakalim-induced vasodilation. Levcromakalim did not significantly alter endothelial nitric oxide synthase phosphorylation, whereas Lipofundin MCT/LCT decreased cyclic guanosine monophosphate. Lipofundin MCT/LCT did not significantly alter levcromakalim-induced membrane hyperpolarization. Taken together, these results suggest that Lipofundin MCT/LCT inhibits the vasodilation induced by levcromakalim by inhibiting basally released endothelial nitric oxide, which seems to occur through medium-chain fatty acids.

## 1. Introduction

Lipid emulsion effectively treats the systemic toxicity induced by toxic doses of local anaesthetics or other drugs with high lipid solubility [1]. The lipid emulsion used for the treatment of local anaesthetic-induced systemic toxicity includes 20% Intralipid, composed of 100% long-chain fatty acids, and 20% Lipofundin MCT/LCT, composed of 50% long-chain fatty acids and 50% medium-chain fatty acids [2]. The underlying mechanisms associated with lipid emulsion treatment include indirect and direct mechanisms [1]. The widely accepted underlying mechanism of lipid emulsion treatment is lipid shuttling; this indirect mechanism occurs when the lipid phase of the lipid emulsion absorbs highly lipid-soluble local anaesthetics such as bupivacaine from the heart, and then, the lipid emulsion containing the sequestered lipid-soluble local anaesthetic (for example, bupivacaine) is transported to the liver and muscle for detoxification and storage [1]. In addition, the direct mechanism of lipid emulsion treatment includes inotropic effects, fatty acid supply, nitric oxide (NO) release inhibition, glycogen synthase kinase-3β phosphorylation, and mitochondrial dysfunction amelioration [1]. As the direct and indirect mechanisms of lipid emulsion treatment overlap, it is very difficult to disentangle the direct and indirect mechanisms of lipid emulsion treatment in an in vivo and in vitro study, which is an important dilemma that laboratory studies regarding lipid emulsion resuscitation must face [3].

NO is involved in ATP-sensitive potassium (K_ATP_) channel-induced vasodilation via cyclic guanosine monophosphate (cGMP) and contributes to membrane hyperpolarization via the K_ATP_ channel [4,5]. In addition, the vasodilation induced by the K_ATP_ channel agonist levcromakalim is augmented by basally released NO [6,7]. However, Intralipid, which increases free fatty acids, enhances blood pressure and systemic vascular resistance, reduces flow-mediated vasodilation, which seems to be associated with impaired NO release due to endothelial dysfunction [8,9,10]. Furthermore, lipid emulsion attenuates the NO-mediated relaxation induced by acetylcholine [11]. This pathophysiological state, including hypotension, acidosis and hypoxia induced by local anaesthetic systemic toxicity, causes K_ATP_ channel-induced vasodilation as a protective response through the attenuation of calcium influx and lipid emulsion has been used to alleviate cardiovascular collapse induced by toxic doses of a local anaesthetic mainly through indirect mechanisms [1,3,12,13]. However, the direct effect of lipid emulsion alone, such as Intralipid and Lipofundin MCT/LCT, on the vasodilation induced by K_ATP_ channels remains to be determined. We hypothesized that lipid emulsion alone attenuates K_ATP_ channel-induced vasodilation via the inhibition of endothelial NO release. The goal of this in vitro study was to compare the effect of Intralipid and Lipofundin MCT/LCT on the vasodilation of isolated rat aortae induced by the K_ATP_ channel agonist levcromakalim and to examine the underlying mechanism with a particular focus on endothelial NO.

## 2. Results

**Figure 1:**
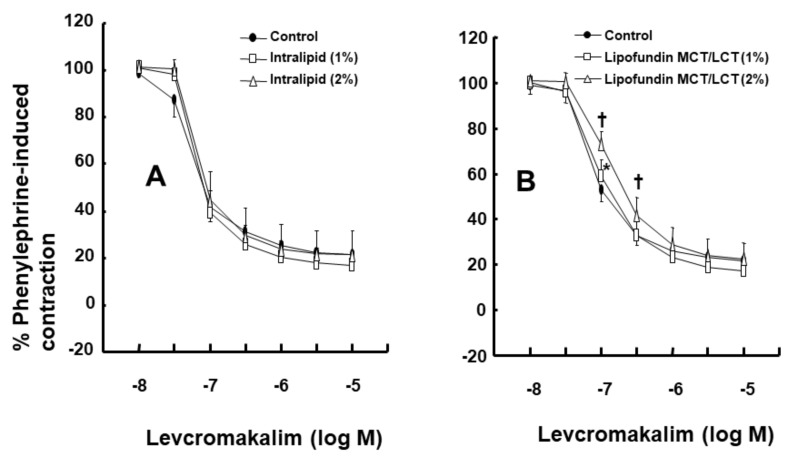
Effect of Intralipid (**A**, *n* = 9) and Lipofundin MCT/LCT (**B**, *n* = 9) on levcromakalim-induced vasodilation in endothelium-intact rat aortae. Data are shown as the mean ± SD and expressed as the percentage of the maximal contraction induced by phenylephrine (10^−6^ M). N indicates the number of isolated rat aortae. * *p* < 0.05 and † *p* < 0.001 versus control.

**Figure 2:**
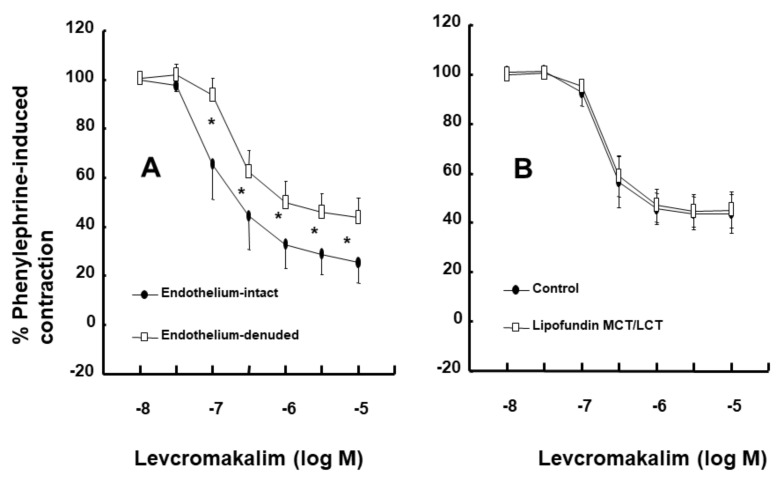
Effect of endothelial denudation (*n* = 13) on the levcromakalim-induced vasodilation of isolated rat aortae (**A**). Effect of Lipofundin MCT/LCT (2%; *n* = 8) on the levcromakalim-induced vasodilation of isolated endothelium-denuded rat aortae (**B**). Data are shown as the mean ± SD and expressed as the percentage of the maximal contraction induced by phenylephrine (10^−6^ M). *n* indicates the number of isolated rat aortae. * *p* < 0.001 versus endothelium-denuded aortae.

**Figure 3:**
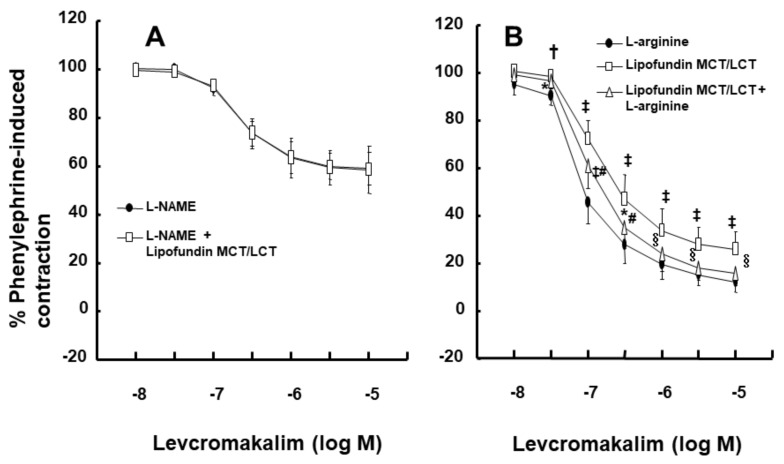
Effect of Lipofundin MCT/LCT (2%, *n* = 8) on the levcromakalim-induced vasodilation of isolated endothelium-intact rat aortae pretreated with N^W^-nitro-L-arginine methyl ester (L-NAME, 10^−4^ M) (**A**). Effect of L-arginine (10^−4^ M) and Lipofundin MCT/LCT (2%), alone (*n* = 9) or combined (*n* = 9), on the levcromakalim-induced vasodilation of isolated endothelium-intact rat aortae (**B**). Data are shown as the mean ± SD and expressed as the percentage of maximal contraction induced by phenylephrine (10^−6^ M). *n* indicates the number of isolated rat aortae. * *p* < 0.05, † *p* < 0.01 and ‡ *p* < 0.001 versus L-arginine alone. § *p* < 0.01 and # *p* < 0.001 versus Lipofundin MCT/LCT alone.

**Figure 4:**
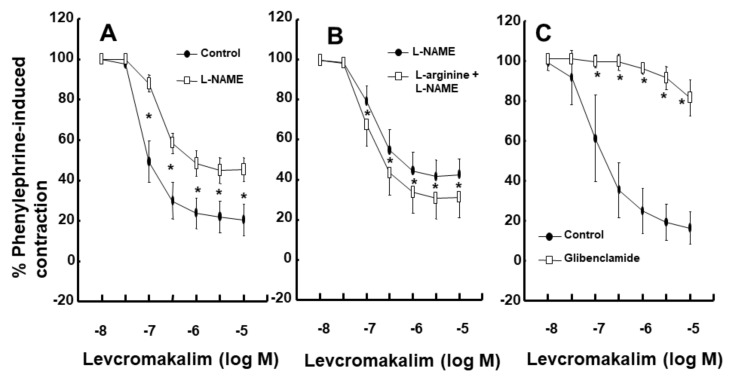
Effect of N^W^-nitro-L-arginine methyl ester (L-NAME, 10^−4^ M, *n* = 8) on levcromakalim-induced vasodilation in isolated endothelium-intact rat aortae (**A**). Effect of L-arginine (10^−4^ M, *n* = 15) alone and effect of combined treatment (M = 15) with L-arginine and L-NAME on the levcromakalim-induced vasodilation of endothelium-intact rat aortae (**B**). Effect of glibenclamide (10^−5^ M, *n* = 8) on the levcromakalim-induced vasodilation of endothelium-intact rat aortae (**C**). Data are shown as the mean ± SD and expressed as the percentage of the maximal contraction induced by phenylephrine (10^−6^ M). *n* indicates the number of isolated rat aortae. A: *p < 0.001 versus control. B: *p < 0.001 versus L-NAME alone. C: * *p* < 0.001 versus control.

**Figure 5:**
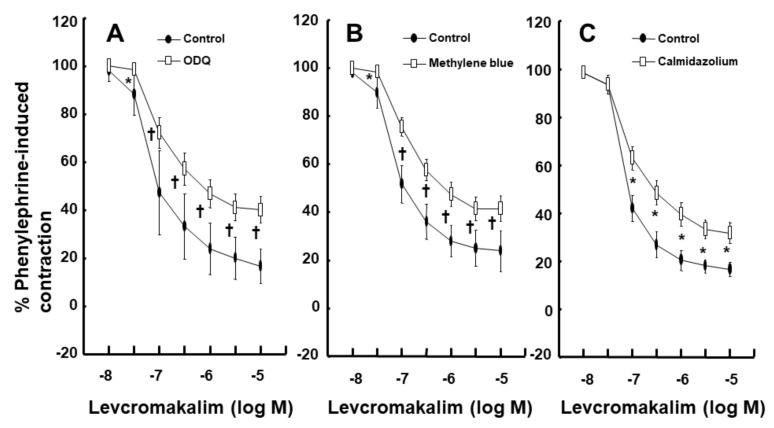
Effect of 1H-[1,2,4]oxadiazolo[4,3-a]quinoxalin-1-one (ODQ, 10^−6^ M, **A**, *n* = 8), methylene blue (10^−6^ M, **B**, *n* = 8) and calmidazolium (3 × 10^−5^ M, **C**, *n* = 8) on the levcromakalim-induced vasodilation of isolated endothelium-intact rat aortae. Data are shown as the mean ± SD and expressed as the percentage of maximal contraction induced by phenylephrine (10^−6^ M). *n* indicates the number of isolated rat aortae. A: * *p* < 0.05 and † *p* < 0.001 versus control. B: * *p* < 0.01 and † *p* < 0.001 versus control. C: * *p* < 0.001 versus control.

**Figure 6:**
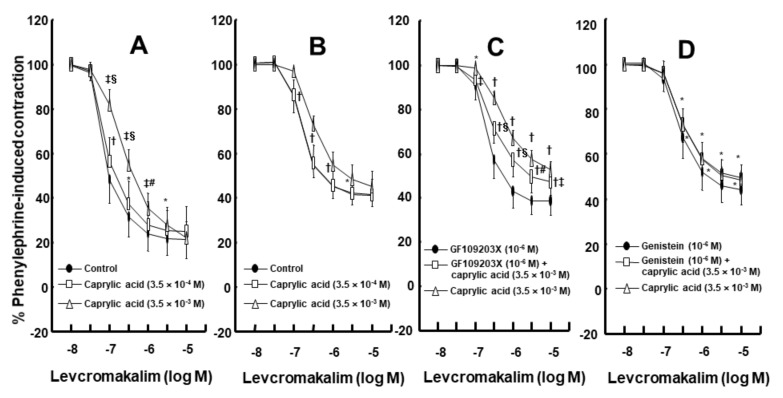
Effect of caprylic acid on the levcromakalim-induced vasodilation of isolated endothelium-intact (**A**; *n* = 12) and endothelium-denuded (**B**; control, 3.5 × 10^−4^ and 3.5 × 10^−3^ caprylic acid, *n*= 8, 7, and 8, respectively.) rat aortae (**A**,**B**). Data are shown as the mean ± SD and expressed as the percentage of the maximal contraction induced by phenylephrine (10^−6^ M). *n* indicates the number of isolated rat aortae. A: * *p* < 0.05, † *p* < 0.01 and ‡ *p*< 0.001 versus control. # *p* < 0.05 and § *p* < 0.001 versus caprylic acid (3.5 × 10^−4^ M). B: * *p* < 0.05, † *p* < 0.001 versus control. Effect of GF109203X, genistein and caprylic acid, alone and combined (**C**: *n* = 8; **D**: *n* = 9), on the levcromakalim-induced vasodilation of endothelium-denuded rat aortae (**C**,**D**). Data are shown as the mean ± SD and expressed as the percentage of maximal contraction induced by phenylephrine (10^−6^ M). *n* indicates the number of isolated rat aortae. C: * *p* < 0.01 and † *p* < 0001 versus GF109203X alone. ‡ *p* < 0.05, # *p* < 0.01 and § *p* < 0.001 versus caprylic acid alone. D: * *p* < 0.001 versus genistein alone.

Intralipid (1% and 2%) did not significantly alter the levcromakalim-induced vasodilation of isolated endothelium-intact rat aortae (Figure 1A). However, Lipofundin MCT/LCT (2%) significantly attenuated the levcromakalim-induced vasodilation of isolated endothelium-intact rat aortae (Figure S1 and Figure 1B; *p* < 0.001 versus control at 10^−7^ and 3 × 10^−7^ M levcromakalim). In addition, Lipofundin MCT/LCT (1%) slightly inhibited levcromakalim (10^−7^ M)-induced vasodilation (*p* < 0.05 versus control; 95% confidence interval: 1.368 to 11.171). Levcromakalim-induced vasodilation was significantly higher in endothelium-intact rat aortae than in endothelium-denuded rat aortae (Figure 2A; *p* < 0.001 at 10^−7^ to 10^−5^ M levcromakalim). Lipofundin MCT/LCT (2%) had no effect on the levcromakalim-induced vasodilation of isolated endothelium-denuded rat aortae (Figure S1 and Figure 2B). In addition, Lipofundin MCT/LCT (2%) had no effect on the levcromakalim-induced vasodilation of isolated endothelium-intact rat aortae pretreated with the nitric oxide synthase (NOS) inhibitor N^W^-nitro-L-arginine methyl ester (L-NAME, 10^−4^ M) (Figure 3A). Furthermore, compared with L-arginine (10^−4^ M) alone, Lipofundin MCT/LCT (2%) significantly inhibited the levcromakalim-induced vasodilation of isolated endothelium-intact rat aortae (Figure 3B; *p* < 0.01 at 3 × 10^−8^ to 10^−5^ M levcromakalim), whereas compared with Lipofundin MCT/LCT (2%) alone, the combined treatment of Lipofundin MCT/LCT (2%) and L-arginine (10^−4^ M) significantly increased the levcromakalim-induced vasodilation of isolated endothelium-intact rat aortae (Figure 3B; *p* < 0.01 versus 10^−7^ to 10^−5^ M levcromakalim). Pre-treatment with L-NAME (10^−4^ M) significantly inhibited the levcromakalim-induced vasodilation of isolated endothelium-intact rat aortae (Figure 4A; *p* < 0.001 versus 10^−7^ to 10^−5^ M levcromakalim). However, the combined treatment with L-arginine (10^−4^ M) and L-NAME (10^−4^ M) significantly increased levcromakalim-induced vasodilation of isolated endothelium-intact rat aortae compared with L-NAME treatment (10^−4^ M) alone (Figure 4B; *p* < 0.001 at 10^−7^ to 10^−5^ M levcromakalim). The K_ATP_ channel inhibitor glibenclamide (10^−5^ M) significantly inhibited the levcromakalim-induced vasodilation (Figure 4C; *p* < 0.001 versus control at 10^−7^ to 10^−5^ M levcromakalim) of endothelium-intact rat aortae. The NO-sensitive guanylate cyclase inhibitor ODQ (10^−6^ M) and non-specific guanylate cyclase inhibitor methylene blue (10^−6^ M) inhibited the levcromakalim-induced vasodilation of isolated endothelium-intact rat aortae (Figure 5A,B; *p* < 0.05 versus control at 3 × 10^−8^ to 10^−5^ M levcromakalim). The calmodulin-regulated enzyme inhibitor calmidazolium (3 × 10^−5^ M) inhibited levcromakalim-induced vasodilation (Figure 5C; *p* < 0.001 versus control at 10^−7^ to 10^−5^ M levcromakalim). The low and high concentrations of caprylic acid (3.5 × 10^−4^ and 3.5 × 10^−3^ M) inhibited the levcromakalim-induced vasodilation of isolated endothelium-intact rat aortae in a concentration-dependent manner (Figure 6A; *p* < 0.05 versus control at 10^−7^ to 10^−6^ M levcromakalim). However, only a high concentration (3.5 × 10^−3^ M) of caprylic acid inhibited the levcromakalim-induced vasodilation of endothelium-denuded rat aortae (Figure 6B; *p* < 0.05 versus control at 10^−7^ and 3 × 10^−6^ M levcromakalim). Compared with caprylic acid (3.5 × 10^−3^ M) treatment alone, the combined treatment of the protein kinase C (PKC) inhibitor GF109203X (10^−6^ M) and caprylic acid (3.5 × 10^−3^ M) significantly increased the levcromakalim-induced vasodilation of endothelium-denuded rat aortae (Figure 6C; *p* < 0.05 at 10^−7^ M to 10^−5^ M levcromakalim). However, the combined treatment of the tyrosine kinase inhibitor genistein (10^−6^ M) and caprylic acid (3.5 × 10^−3^ M) had no effect on the levcromakalim-induced vasodilation of endothelium-denuded rat aortae compared with caprylic acid (3.5 × 10^−3^ M) treatment alone (Figure 6D).

**Figure 7:**
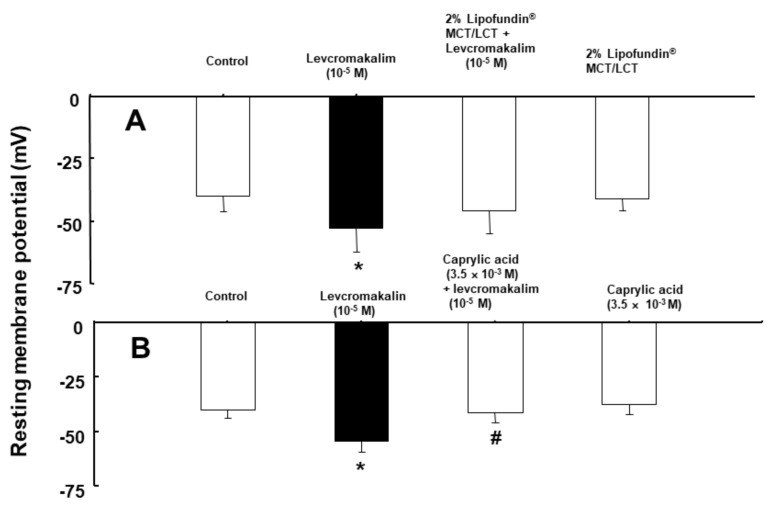
Effect of Lipofundin MCT/LCT (**A**; *n* = 6~8) and caprylic acid (**B**; *n* = 8) on levcromakalim-induced membrane hyperpolarization in rat vascular smooth muscle cells. Membrane potentials were analysed in two different sets of experiments. Each bar is the mean ± SD of three repeated experiments. A: * *p* < 0.05 versus control. B: * *p* < 0.001 versus control. # *p* < 0.001 versus levcromakalim alone.

Levcromakalim (10^−5^ M) induced membrane hyperpolarization in rat aortic vascular smooth muscle cells (VSMCs) (Figure 7A; *p* < 0.05 versus control; control: −39.9 ± 6.3 mV versus levcromakalim: −52.8 ± 9.7 mV). Lipofundin MCT/LCT (2%) did not significantly alter levcromakalim-induced membrane hyperpolarization (Figure 7A). However, a high concentration of caprylic acid (3.5 × 10^−3^ M) significantly inhibited the levcromakalim (10^−5^ M)-induced membrane hyperpolarization of rat aortic VSMCs (Figure 7B; *p* < 0.001 versus levcromakalim alone; levcromakalim: −54.3 ± 4.9 mV versus caprylic acid plus levcromakalim: −41.2 ± 4.3 mV). The potential values were corrected for a + 2 mV liquid junction potential at the 3M KCl/agar salt bridge. A change in membrane potential appeared within 1 min after treatment with chemicals.

**Figure 8:**
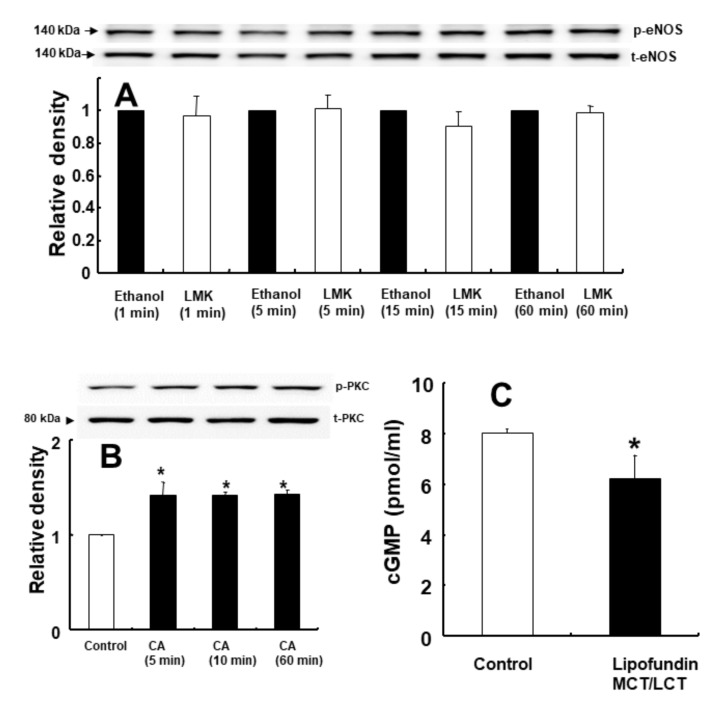
Effect of levcromakalim (LMK) on endothelial nitric oxide synthase (eNOS) phosphorylation (at Ser 1177) in human umbilical vein endothelial cells (HUVECs) (**A**). HUVECs were treated with LMK (10^−5^ M) for 1, 5, 15 and 60 min. Data (*n* = 3) are shown as the mean ± SD. *p*-eNOS: phosphorylated eNOS; t-eNOS: total eNOS. Effect of caprylic acid (CA; 3.5 × 10^−3^ M; *n* = 3) on protein kinase C (PKC) phosphorylation in rat aortic vascular smooth muscle cells (VSMCs) (**B**). VSMCs were treated with CA for 5, 10 and 60 min. Data are shown as the mean ± SD. *p < 0.001 versus control. *p*-PKC: phosphorylated PKC. t-PKC: total PKC. Effect of Lipofundin MCT/LCT (2%; *n* = 3) on cyclic guanosine monophosphate (cGMP) formation in isolated endothelium-intact rat aortae (**C)**. Data are shown as the mean ± SD. *n* indicates the number of rats. *p < 0.05 versus control.

Levcromakalim (10^−5^ M) did not significantly alter the phosphorylation of endothelial nitric oxide synthase (eNOS) in human umbilical vein endothelial cells (HUVECs) (Figure 8A). A high concentration of caprylic acid (3.5 × 10^−3^ M) induced PKC phosphorylation in rat aortic VSMCs (Figure 8B: *p* < 0.001 versus control). Lipofundin MCT/LCT (2%) decreased cGMP formation in endothelium-intact rat aortae (Figure 8C: *p* < 0.05 versus control).

## 3. Discussion

This study indicates that the medium-chain fatty acids contained in Lipofundin MCT/LCT inhibit levcromakalim-induced vasodilation via the inhibition of endothelial NO release. The major findings of this study are as follows: (1) Lipofundin MCT/LCT inhibited levcromakalim-induced vasodilation, whereas Intralipid did not significantly change levcromakalim-induced vasodilation; (2) L-arginine attenuated the Lipofundin MCT/LCT-mediated inhibition of levcromakalim-induced vasodilation of endothelium-intact rat aorta; (3) Lipofundin MCT/LCT did not significantly change the levcromakalim-induced membrane hyperpolarization of VSMCs; and 4) Levcromakalim did not significantly alter eNOS phosphorylation, whereas Lipofundin MCT/LCT decreased cGMP formation.

Intralipid contains 100% long-chain fatty acids, namely, 53% linoleic acid, 24% oleic acid, 11% palmitic acid, 8% alpha-linolenic acid and 4% stearic acid, whereas Lipofundin MCT/LCT contains 50% long-chain fatty acids and 50% medium-chain fatty acids [2]. The medium-chain fatty acids in Lipofundin MCT/LCT are as follows: 28.5% caprylic acid, 20% capric acid, 1% lauric acid, and 0.5% caproic acid [2]. Similar to previous reports indicating that the magnitude of the Lipofundin MCT/LCT-mediated inhibition of vasodilation induced by acetylcholine via NO was higher than that of Intralipid and that Lipofundin MCT/LCT only increased blood pressure, Lipofundin MCT/LCT only inhibited the levcromakalim-induced vasodilation of isolated endothelium-intact rat aortae (Figure 1B) [11,14]. However, Lipofundin MCT/LCT (2%) had no effect on the levcromakalim-induced vasodilation of isolated endothelium-denuded rat aortae (Figure 2B) and endothelium-intact rat aortae pretreated with L-NAME (Figure 3A). Moreover, Lipofundin MCT/LCT decreased cGMP formation (Figure 8C). In agreement with a previous report that basally released NO enhanced the levcromakalim-induced vasodilation of endothelium-intact rat aortae, levcromakalim-induced vasodilation was enhanced in endothelium-intact rat aortae (Figure 2A) [6]. Taken together with previous reports, these results suggest that the inhibitory effect of Lipofundin MCT/LCT on levcromakalim-induced vasodilation is due to the inhibition of basally released NO-mediated K_ATP_ channel-induced vasodilation by medium-chain fatty acids [2,6,7]. Supporting the above comment, L-arginine, which is a substrate of NOS, attenuated the Lipofundin MCT/LCT-mediated inhibition of levcromakalim-induced vasodilation (Figure 3B), suggesting that Lipofundin MCT/LCT acts as an NOS inhibitor similar to L-NAME. In agreement with the current results and a previous report, Lipofundin MCT/LCT did not significantly alter the levcromakalim-induced membrane hyperpolarization of VSMCs (Figure 7A) [5]. Thus, further study on which medium-chain fatty acid contained in Lipofundin MCT/LCT is mainly involved in the inhibition of levcromakalim-induced vasodilation via the attenuation of NO release is needed. Low (3.5 × 10^−4^ M) and high (3.5 × 10^−3^ M) concentrations of caprylic acid, which are approximately 48 and 4.8 times lower than that contained in 1% Lipofundin MCT/LCT (from B. Brown), respectively, inhibited the levcromakalim-induced vasodilation of endothelium-intact rat aortae in a concentration-dependent manner (Figure 6A). However, a high concentration (3.5 × 10^−3^ M) of caprylic acid only inhibited the levcromakalim-induced vasodilation of endothelium-denuded rat aortae (Figure 6B). Caprylic acid (C8:0) initially induces depolarization in rat pancreatic β-cells in the presence of glucose, which is associated with inhibition of K_ATP_ channels [15]. Similar to this report and consistent with the results of the current tension study, a high concentration of caprylic acid (3.5 × 10^−3^ M) inhibited the levcromakalim-induced membrane hyperpolarization of VSMCs (Figure 7B) [15]. In addition, similar to a previous report, the PKC inhibitor GF109023X attenuated the caprylic acid-mediated inhibition of the levcromakalim-induced vasodilation (Figure 6C) of endothelium-denuded rat aortae and the caprylic acid induced PKC phosphorylation (Figure 8B) [16]. In contrast, the tyrosine kinase inhibitor genistein had no effect on the Lipofundin MCT/LCT-mediated inhibition of levcromakalim-induced vasodilation (Figure 6D). Taken together, these results suggest that inhibition of levcromakalim-induced vasodilation by high concentrations of caprylic acid is mediated by the effect of PKC activation in VSMCs.

Calcium-dependent eNOS, which is activated by calmodulin, produces NO from L-arginine, which is a substrate of eNOS [17]. NO is moved from the endothelium to vascular smooth muscle [17]. Then, the NO in vascular smooth muscle activates guanylate cyclase to produce cGMP [17]. Activated cGMP induces vasodilation via cGMP-dependent protein kinase activation [17]. Basally released NO-mediated cGMP-dependent protein kinase activation produces vasodilation via the activation of the sulfonylurea receptor of K_ATP_ channels, which is composed of an inward rectifying potassium channel (Kir 61 or Kir 6.2) and regulatory sulfonylurea receptor [7]. Consistent with previous reports, the calmodulin-regulated enzyme inhibitor calmidazolium and the NOS inhibitor L-NAME inhibited levcromakalim-induced vasodilation (Figure 4A and Figure 5C) [6,7]. In addition, the NO-sensitive guanylate cyclase inhibitor ODQ and the nonspecific guanylate cyclase inhibitor methylene blue attenuated levcromakalim-induced vasodilation (Figure 5A,B). Furthermore, L-arginine, which is both a substrate of NOS and competitive inhibitor of the NOS inhibitor L-NAME, increased the levcromakalim-induced vasodilation of endothelium-intact rat aortae pretreated with L-NAME (Figure 4B) [18]. Taking into consideration previous reports, as glibenclamide nearly abolished the levcromakalim-induced vasodilation of endothelium-intact aortae (Figure 4C) and as levcromakalim alone did not significantly alter eNOS phosphorylation (Figure 8A), the levcromakalim-induced vasodilation of endothelium-intact aortae is augmented by basally released endothelial NO-mediated K_ATP_ channel activation, which is mediated by a pathway involving calmodulin, eNOS, cGMP and K_ATP_ channels [4,5,6,7].

Intralipid is mainly used to treat local anaesthetic systemic toxicity, and Lipofundin MCT/LCT is sometimes used to treat local anaesthetic systemic toxicity [19,20,21,22,23,24]. Although lipid emulsion alleviates the toxic dose of bupivacaine-mediated inhibition of vasodilation induced by the K_ATP_ channel agonist levcromakalim primarily by scavenging effects regarded as indirect action, Intralipid may provide a more favourable effect than Lipofundin MCT/LCT on the maintenance of vasodilation induced by K_ATP_ channel activation mediated through the pathophysiological state caused by local anaesthetic systemic toxicity [25]. However, this study has some limitations. First, organ blood flow is regulated by small resistance arterioles, whereas rat aortae, which are regarded as conduit vessels, were used in the current study [26]. Second, in an in vivo study, lipid emulsion alone increased left ventricular systolic pressure via the inhibition of NO release and increased blood pressure, which is one of the proposed underlying mechanisms of lipid emulsion treatment; however, this study was an in vitro study that did not consider the regulation of organ blood flow by the heart and sympathetic nervous system [8,9,10,27]. Third, we did not examine the effect of several other medium-chain free fatty acids other than caprylic acid contained in Lipofundin MCT/LCT on levcromakalim-induced vasodilation [2]. Fourth, the pathophysiological state, including hypoxia, acidosis and hypotension, induces K_ATP_ channel activation in vivo [12]. However, we used the K_ATP_ channel agonist levcromakalim to activate K_ATP_ channels in the current experiment. These two states (endogenous pathophysiological state-induced K_ATP_ channel activation versus levcromakalim-induced K_ATP_ channel activation) may be different [7].

## 4. Materials and Methods

The experimental protocol (GNU-160414-R0019, 6 April 2016) was approved by the Institutional Animal Care and Use Committee of Gyeongsang National University. All experimental procedures were performed following the guidelines of the Care and Use of Laboratory Animals of Gyeongsang National University.

### 4.1. Preparation of Isolated Rat Aorta and Isometric Tension Measurement

Male Sprague-Dawley rats (body weight: 240–300 g) were anaesthetized by 100% carbon dioxide supplied via a small hole in the rat cage. Isolated rat aortae to be used for isometric tension measurement were prepared as described in a previous study [28]. The rat thoracic cavity was exposed, and the descending thoracic rat aorta was removed from the thoracic cavity. Descending thoracic rat aortae were immersed in Krebs solution, which included sodium chloride (118 mM), sodium bicarbonate (25 mM), glucose (11 mM), potassium chloride (4.7 mM), calcium chloride (2.4 mM), magnesium sulfate (1.2 mM), and monopotassium phosphate (1.2 mM). We removed the perivascular tissues, including connective tissue and fat, surrounding isolated descending thoracic rat aortae immersed in Krebs solution under a microscope. The isolated descending thoracic aorta was cut into 2.5-mm segments of aortic rings. The endothelium of some thoracic aortic rings was removed by inserting two 25-gauge needles into the lumen of aortic rings and rolling the aortic ring backward and forward. The isolated thoracic aortic rings were suspended in a Grass isometric transducer (FT-03, Grass Instrument, Quincy, Massachusetts, USA) attached to an organ bath filled with 10 cc Krebs solution maintained at 37 °C. Based on the previous experiment, a baseline resting tension of 2.5 g was chosen and maintained for 120 min to reach equilibrium of 2.5 g [29]. While baseline resting tension was maintained for 120 min, the Krebs solution was exchanged with fresh Krebs solution every 40 min. The pH of the Krebs solution was maintained at 7.4 by aerating the Krebs solution with 95% oxygen and 5% carbon dioxide. The integrity of the endothelium from endothelium-intact rat aortae was confirmed as follows. Phenylephrine (10^−7^ M) induced a sustained and stable contraction, and then acetylcholine (10^−5^ M) was added into the organ bath. An aorta with more than 85% acetylcholine-induced relaxation from the phenylephrine-induced contraction was regarded as an endothelium-intact rat aorta. The removal of the endothelium from endothelium-denuded rat aortae was confirmed as follows. Phenylephrine (10^−8^ M) induced a sustained and stable contraction, and then acetylcholine (10^−5^ M) was added into the organ bath. An aorta with less than 15% acetylcholine-induced relaxation was regarded as an endothelium-denuded rat aorta. The isolated rat aortae with or without endothelium showing acetylcholine-induced relaxation were washed with fresh Krebs solution several times to restore the baseline resting tension. After the baseline resting tension was restored, the following experimental protocols were performed.

### 4.2. Experimental Protocol

First, the effects of Intralipid with 100% long-chain fatty acids and Lipofundin MCT/LCT with 50% long-chain fatty acids and 50% medium-chain fatty acids on the vasodilation induced by levcromakalim in the isolated endothelium-intact rat aorta were examined. The endothelium-intact rat aortae were pretreated with Intralipid (1 and 2%) or Lipofundin MCT/LCT (1% and 2%) for 20 min. After phenylephrine (10^−6^ M) produced vasoconstriction in the endothelium-intact rat aorta in the presence or absence of Intralipid or Lipofundin MCT/LCT, levcromakalim (10^−8^ to 10^−5^ M) was cumulatively added into the organ bath to produce levcromakalim concentration-response curves.

Second, the effects of endothelial denudation on the vasodilation induced by levcromakalim were examined. In addition, the effect of Lipofundin MCT/LCT on the vasodilation induced by levcromakalim in isolated endothelium-denuded rat aortae was examined. The endothelium-denuded rat aortae were pretreated with Lipofundin MCT/LCT (2%) for 20 min. After phenylephrine (10^−6^ M) produced a sustained and stable contraction in the isolated endothelium-denuded rat aorta in the presence or absence of Lipofundin MCT/LCT (2%) or in the isolated rat aorta with or without endothelium, levcromakalim (10^−8^ to 10^−5^ M) was cumulatively added into the organ bath to produce levcromakalim concentration-response curves.

Third, the effects of the NOS inhibitor L-NAME, and L-arginine, which is both substrates for NOS and competitive inhibitors of NOS inhibitors such as L-NAME, on the Lipofundin MCT/LCT-mediated inhibition of the levcromakalim-induced vasodilation of endothelium-intact rat aortae were examined. The endothelium-intact rat aortae were pre-treated with L-NAME (10^−4^ M) for 20 min followed by Lipofundin MCT/LCT (2%) for 15 min, L-NAME (10^−4^ M) alone for 35 min, L-arginine (10^−4^ M) and Lipofundin MCT/LCT (2%) alone for 30 min, and Lipofundin MCT/LCT (2%) plus L-arginine (10^−4^ M) for 30 min. After phenylephrine (10^−6^ M) produced a sustained and stable contraction in the presence or absence of L-NAME, L-arginine, or Lipofundin MCT/LCT alone or in the presence of combined treatment with either L-NAME plus Lipofundin MCT/LCT or Lipofundin MCT/LCT plus L-arginine, levcromakalim (10^−8^ to 10^−5^ M) was cumulatively added into organ bath to produce levcromakalim-concentration curves.

Fourth, the effects of L-NAME and the K_ATP_ channel inhibitor glibenclamide alone and of combined treatment with L-arginine and L-NAME on the vasodilation induced by levcromakalim in isolated endothelium-intact rat aortae were examined. The endothelium-intact rat aortae were pretreated with L-NAME (10^−4^ M) alone or L-arginine (10^−4^ M) plus L-NAME (10^−4^ M) for 30 min [6]. In addition, endothelium-intact rat aortae were treated with indomethacin (10^−5^ M) alone for 35 min or indomethacin (10^−5^ M) for 15 min followed by glibenclamide (10^−5^ M) for 20 min [6]. After phenylephrine (10^−6^ M) produced a sustained and stable contraction in the presence or absence of L-NAME and glibenclamide alone or in the presence of combined treatment with L-arginine and L-NAME, levcromakalim (10^−8^ to 10^−5^ M) was cumulatively added into the organ bath to produce levcromakalim concentration-response curves.

Fifth, the effects of the NO-sensitive guanylate cyclase inhibitor 1H-[1,2,4]oxadiazolo[4,3-a]quinoxalin-1-one (ODQ), the non-specific guanylate cyclase inhibitor methylene blue and the calmodulin-regulated enzyme inhibitor calmidazolium on the vasodilation of endothelium-intact rat aortae induced by levcromakalim were examined. The endothelium-intact rat aortae were pretreated with inhibitor alone (10^−6^ M ODQ, 10^−6^ M methylene blue or 3 × 10^−5^ M calmidazolium) for 20 min [6]. After phenylephrine (10^−6^ M) produced a sustained and stable contraction in the endothelium-intact rat aorta in the presence or absence of each inhibitor, levcromakalim (10^−8^ to 10^−5^ M) was cumulatively added into the organ bath to produce levcromakalim concentration-response curves.

Sixth, the effects of caprylic acid, which is one of the main medium-chain fatty acids contained in Lipofundin MCT/LCT, on the vasodilation induced by levcromakalim in endothelium-intact and endothelium-denuded rat aortae were examined. The endothelium-intact and endothelium-denuded rat aortae were pretreated with caprylic acid (3.5 × 10^−4^ and 3.5 × 10^−3^ M) for 20 min. After phenylephrine (10^−6^ M) produced a sustained and stable contraction in isolated endothelium-intact and endothelium-denuded aortae in the presence or absence of caprylic acid, levcromakalim (10^−8^ to 10^−5^ M) was cumulatively added into the organ bath to produce levcromakalim concentration-response curves. In addition, as the activation of PKC or tyrosine kinase inhibits the vasodilation induced by K_ATP_ channels, the effects of the PKC inhibitor GF109023X and the tyrosine kinase inhibitor genistein on the vasodilation induced by levcromakalim in isolated endothelium-denuded rat aortae with caprylic acid were examined [16,30]. The endothelium-denuded rat aortae were pretreated with GF109203X (10^−6^ M) or genistein (10^−6^ M) alone for 35 min, caprylic acid (3.5 × 10^−3^ M) alone for 20 min and GF109203X (10^−6^ M) or genistein (10^−6^ M) for 15 min followed by caprylic acid (3.5 × 10^−3^ M) for 20 min. After phenylephrine (10^−6^ M) produced a sustained and stable contraction in the presence or absence of the inhibitors and caprylic acid, alone or combined, levcromakalim (10^−8^ to 10^−5^ M) was cumulatively added into the organ bath to produce levcromakalim concentration-response curves.

### 4.3. Cell Culture

VSMCs isolated from rat thoracic aortae and HUVECs (EA.hy926 cells, American Type Culture Collection, Manassas, VA, USA) were cultured in Dulbecco’s modified Eagle’s medium (HyClone, GE Healthcare, UT, USA) supplemented with 10% foetal bovine serum (Gibco, Life Technologies, NY, USA), 100 U/mL penicillin, and 100 µg/mL streptomycin, as previously described [31]. Cells were grown at 37 °C in a humidified atmosphere of 5% CO_2_, and the medium was changed every 2 days until the cells reached confluence. Upon reaching confluence, the cells were harvested using 0.025% trypsin solution and diluted to a 1:4 ratio. For our experiments, cells were used between passages 3 and 5 (VSMCs) or between passages 5 and 9 (HUVECs). Confluent cells were further incubated with serum-free medium overnight prior to drug treatment.

### 4.4. Measuring Membrane Potential

The membrane potential in VSMCs cultured on poly-L-lysine-coated glass coverslips was recorded under current-clamp mode (*I =* 0) using the whole-cell patch clamp technique as described previously [25]. Membrane potentials were amplified with a patch clamp amplifier (Axopatch 200B, Axon Instruments, Union City, CA, USA). A 3 M KCl/agar salt bridge was used to minimize liquid junction potential. The experimental solutions contained the following (mM): 135 NaCl, 5 KCl, 1 CaCl_2_, 1 MgCl_2_, 5 glucose, and 10 HEPES for the bath solution and 150 KCl, 1 MgCl_2_, 5 EGTA, and 10 HEPES for the pipette solution (pH 7.3). The resistance of the pipette tip was 4-6 MΩ. The pH of the bath and pipette solutions was adjusted to 7.4 and 7.3, respectively, with HCl or KOH. Chemicals were dissolved in the bath solutions at desired concentrations. All experiments were performed at room temperature. The data were analysed using Clampfit (pCLAMP, version 9.2, Axon Instruments) and OriginPro^®^ 2020 (OriginLab Corp. Northampton, MA, USA).

### 4.5. Western Blot

Western blot analysis was performed using the method described by Lee et al. [31]. Cells were prepared in ice-cold RIPA lysis buffer (Cell Signaling Technology, Beverly, MA, USA) containing protease inhibitor cocktail (Thermo Fisher Scientific, Rockford, IL, USA) and phosphatase inhibitor cocktail (Thermo Fisher Scientific). The lysates were centrifuged at 20,000× *g* for 15 min at 4 °C, the supernatants were collected, and the protein concentration was determined using the Bio-Rad protein assay kit (Bio-Rad, Hercules, CA, USA). Total protein (30 µg) was denatured by heating at 100 °C for 10 min and then separated by 8–10% sodium dodecyl sulfate-polyacrylamide gel electrophoresis. The separated proteins were transferred onto polyvinylidene difluoride membranes (Millipore, Bedford, MA, USA). After blocking with 5% bovine serum albumin or 5% skim milk at room temperature for 1 h, the membranes were incubated with primary antibodies (anti-phospho-eNOS at Ser1177 [1:1000], anti-eNOS [1:1000], anti-β-actin [1:10000], anti-phospho-PKC [1:1000] and anti-PKC [1:500]) at 4 °C overnight. After washing with Tris-buffered saline containing 0.1% Tween-20 (TBST), the membranes were incubated with a 1:5000 dilution of the corresponding anti-rabbit or anti-mouse IgG secondary antibody for 1 h at room temperature. After washing three times with TBST, immune complexes were visualized with SuperSignal^®^ West Pico Chemiluminescent Substrate (Thermo Scientific). The density values of the bands were quantified using Image Lab software v.3.0 (Bio-Rad Laboratories, Inc., Hercules, CA, USA).

### 4.6. cGMP Measurement

The cGMP measurement was performed following methods described previously [28]. The descending thoracic aortic strips were immersed in Krebs solution contained in a 10-cc organ bath for 60 min. Endothelium-intact thoracic aortic strips from the same rat aorta were treated with Lipofundin MCT/LCT (2%) for 45 min. Then, aortic strips were frozen in liquid nitrogen and homogenized in 0.1 M HCl. The acidic supernatants were used, and cGMP was measured by ELISA using the cGMP Complete Kit supplied by Abcam (Cambridge Science Park, Cambridge, England). The amounts of cGMP in each strip were expressed as pmol/mL.

### 4.7. Materials

Lipofundin MCT/LCT and Intralipid were supplied by B. Brown Korea (Seoul, Korea) and Fresenius Kabi Korea (Seoul, Korea), respectively. L-arginine, L-NAME, glibenclamide, ODQ, methylene blue, calmidazolium, indomethacin, caprylic acid, GF109203X and genistein were obtained from Sigma-Aldrich (St Louis, MO, USA). Levcromakalim was purchased from Tocris Bioscience (Bristol, United Kingdom). The anti-phospho-eNOS (at Ser1177), anti-eNOS, and anti-phospho-PKC antibodies were purchased from Cell Signaling Technology (Beverly, MA, USA). Anti-PKC antibody was purchased from Santa Cruz Biotechnology (Santa Cruz, CA, USA). All the chemical concentrations are expressed as the final organ bath concentration. Levcromakalim was dissolved in ethanol (final concentration: 0.19%). Glibenclamide, ODQ, calmidazolium, indomethacin and GF109203X were dissolved in dimethyl sulfoxide (final concentration of dimethyl sulfoxide: less than 0.1%). All other chemicals were dissolved in distilled water.

### 4.8. Statistical Analysis

Data are shown as the mean ± SD. Levcromakalim-induced vasodilation is expressed as the percentage of the maximal contraction induced by phenylephrine. The effects of lipid emulsion, various inhibitors, caprylic acid and endothelial denudation, alone and combined, on levcromakalim-induced vasodilation were analysed using a generalized linear mixed-effect model (Stata version 14.1, StataCorp LP, Lakeway Drive College Station, TX, USA) [32]. The effect of lipid emulsion and caprylic acid on levcromakalim-induced membrane hyperpolarization was analysed using one-way analysis of variance followed by Bonferroni’s post hoc test. The effects of caprylic acid on PKC phosphorylation were analysed using unpaired Student’s t-test. The effects of levcromakalim and lipid emulsion on eNOS phosphorylation and cGMP formation, respectively, were analysed using unpaired Student’s t-test. *p* values less than 0.05 were considered statistically significant.

## 5. Conclusions

Taken together, these results suggest that medium-chain fatty acids contained in Lipofundin MCT/LCT inhibit the vasodilation induced by K_ATP_ channels via the inhibition of basal endothelial NO release. In addition, caprylic acid inhibited levcromakalim-induced vasodilation via the activation of PKC in vascular smooth muscle. The results of this study help to elucidate the mechanism causing lipid emulsion resuscitation in local anaesthetic toxicity.

## Supplementary Materials

Supplementary materials can be found at https://www.mdpi.com/1422-0067/21/5/1763/s1. Figure S1. Raw traces showing the effect of 2% Lipofudin MCT/LCT on the levcromakalim-induced vasodilation in the endothelium-intact and endothelium-denuded rat aortae.

## Abbreviations

K_ATP_ channel	ATP-sensitive potassium channel
VSMC	Vascular smooth muscle cell
NO	Nitric oxide
PKC	Protein kinase C
HUVEC	Human umbilical vein endothelial cell
cGMP	Cyclic guanosine monophosphate
L-NAME	N^W^-nitro-L-arginine methyl ester
