# Supplementary material for: Lipofundin MCT/LCT Inhibits Levcromakalim-Induced Vasodilation by Inhibiting Endothelial Nitric Oxide Release
Source: Int J Mol Sci. 2020 Mar 4;21(5):1763. doi: 10.3390/ijms21051763 (PMC7084418; doi:10.3390/ijms21051763)
Supplement: Supplementary file 1 [file ijms-21-01763-s001.pdf]

International Journal of
Molecular Sciences

MDPI

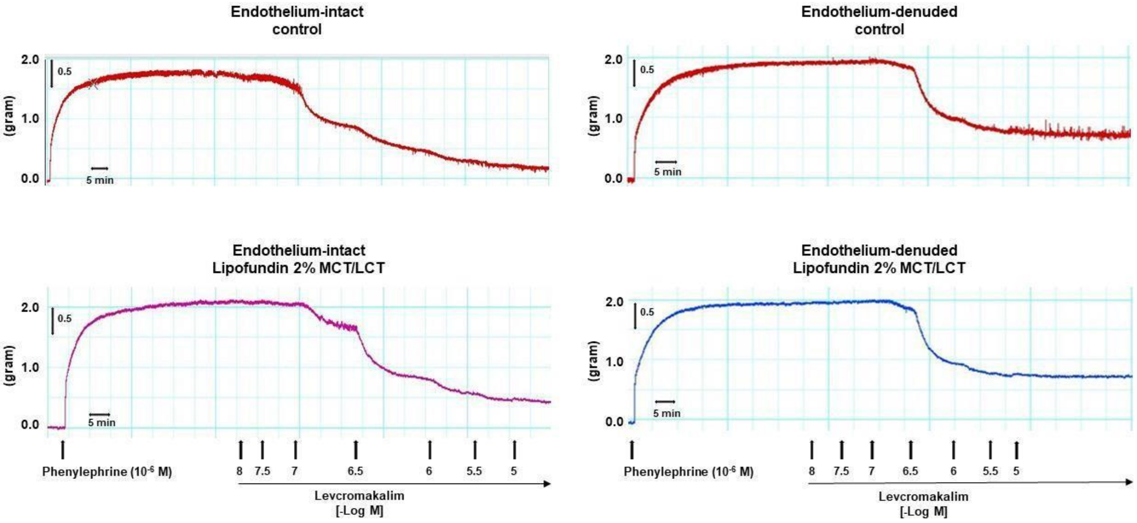

Figure S1. Raw traces showing the effect of 2% Lipofudin MCT/LCT on the levcromakalim-induced vasodilation in the endothelium-intact and endothelium-denuded rat aortae.
